# Online Leisure Activities for Sustained Mental Health Well-being in Older Adults with COVID-19 Mitigation

**DOI:** 10.1093/geroni/igab046.2684

**Published:** 2021-12-17

**Authors:** Solymar Rivera-Torres, Elias Mpofu, M Jean Keller, Stan Ingman

**Affiliations:** 1 University of North Texas, Denton, University of North Texas, Denton, Texas, United States; 2 University of North Texas, University of North Texas, Denton, Texas, United States

## Abstract

Older adults (OA) experience psychosocial distress from the COVID-19 pandemic mitigations. While their participation in leisure and recreation activities (LRA) would be ameliorating, we do not know how LRA OA engages for their mental health (MH) well-being with COVID-19 mitigation. This scoping review aimed to trend the evidence on the types of LRA OA engage for their MH well-being across the young-old continuum (60-69 years) through to older-old (80 years and above) in the COVID-19 pandemic. We searched the following electronic databases (PubMed, EMBASE, CINAHL, Cochrane, JBI-ES, and Epistemonicos for LRA studies by OA with COVID-19 mitigation. To be included, we considered empirical articles published in English on LRA of OA 55+ years-old. Another criterion required articles describing those activities' qualities and the impact of LRA on MH and well-being during the COVID-19 pandemic. We resulted in seven empirical studies, two of which implemented in the USA and one from the USA and Canada, Spain, Israel, and Japan. Findings following narrative synthesis revealed trending evidence on OA to engage in online LRA for social, cognitive /intellectual, and emotional health. Leisure-time physical activity reduced negative MH symptoms as anxiety and depression in OA under COVID-19 threat. In conclusion, the present review's trending evidence suggests that OA engagement in social, physical, mental, and cognitive LRA enhanced their MH and overall well-being. Activities delivered by way of the Internet and television provided a cluster of beneficial opportunities for the OA mental health needs under the COVID-19 pandemic.

